# A novel strategy for treating cancer: understanding the role of Ca^2+^ signaling from nociceptive TRP channels in regulating cancer progression

**DOI:** 10.37349/etat.2021.00053

**Published:** 2021-10-31

**Authors:** Wen-Li Hsu, Mami Noda, Tohru Yoshioka, Etsuro Ito

**Affiliations:** 1 Department of Dermatology, Kaohsiung Municipal Ta-Tung Hospital, Kaohsiung Medical University Hospital, Kaohsiung Medical University, Kaohsiung 80145, Taiwan; 2 Regenerative Medicine and Cell Therapy Research Center, Kaohsiung Medical University, Kaohsiung 80708, Taiwan; 3 Laboratory of Pathophysiology, Graduate School of Pharmaceutical Sciences, Kyushu University, Fukuoka 812-8582, Japan; 4 Graduate Institute of Medicine, School of Medicine, Kaohsiung Medical University, Kaohsiung 80708, Taiwan; 5 Waseda Research Institute for Science and Engineering, Waseda University, Tokyo 162-8480, Japan; 6 Department of Biology, Waseda University, Tokyo 162-8480, Japan; The University of Texas at Arlington, USA

**Keywords:** Aging, nociceptive transient receptor potential channel, cancer progression

## Abstract

Cancer is an aging-associated disease and caused by genomic instability that is driven by the accumulation of mutations and epimutations in the aging process. Although Ca^2+^ signaling, reactive oxygen species (ROS) accumulation, DNA damage response (DDR) and senescence inflammation response (SIR) are processed during genomic instability, the underlying mechanism for the cause of genomic instability and cancer development is still poorly understood and needs to be investigated. Nociceptive transient receptor potential (TRP) channels, which firstly respond to environmental stimuli, such as microbes, chemicals or physical injuries, potentiate regulation of the aging process by Ca^2+^ signaling. In this review, the authors provide an explanation of the dual role of nociceptive TRP channels in regulating cancer progression, initiating cancer progression by aging-induced genomic instability, and promoting malignancy by epigenetic regulation. Thus, therapeutically targeting nociceptive TRP channels seems to be a novel strategy for treating cancers.

## Introduction

Cancer is a genomic disease. Increased rates of cancer in an aging population are an integral component of aging associated diseases [1]. Because genomic instability is caused by the accumulation of mutations and epimutations in the aging process, it contributes significantly to activation of oncogenes and dysfunction in tumor suppressor genes, which are involved in cancer development [2]. Genomic instability derives from DNA damage response (DDR), and the p53 family proteins drive the DNA repair system to recover errors caused by genomic instability in order to maintain homeostasis of normal tissue [3]. Once there is a blockage of the balance between genomic instability and the DNA repair system, DDR results in p53 family proteins dysfunction which promotes cancer progression [4].

Genomic instability is the most important condition to induce cancer development, but it may trigger the key process in initiating cancer progression before activation of oncogenes and dysfunction in tumor suppressor genes. Based on the mitochondrial free radical theory of aging, excessive increase in intracellular reactive oxygen species (ROS) levels induces genomic instability and would lead to cellular dysfunctions and aging [5]. Intracellular ROS accumulation can be stimulated by mitochondria Ca^2+^ overload [6]. Therefore, Ca^2+^ signaling plays an important role in determining intracellular ROS accumulation, DDR, and genomic instability following initiation of cancer progression.

Ca^2+^ signaling is also a crucial regulator of pathways in promoting cancer progression with oncogenic activation [7]. The contribution of Ca^2+^ signaling to cancer cell growth, metastasis and chemotherapy resistance has been extensively investigated [8, 9], including regulation by epigenetic mechanisms to induce malignancy [10]. The therapeutic targeting of Ca^2+^ signaling provides a novel approach for treating cancer. Recently, nociceptive transient receptor potential (TRP) channels, which belong to a special group of TRP channels, have been involved in the nociceptive pathway and include members of the TRP ankyrin (TRPA), and TRP canonical (TRPC), TRP mucolipin (TRPM) and TRP vanilloid (TRPV) subfamilies that potentiate regulation of the aging process and tumorigenesis by Ca^2+^ signaling [11–15]. This is different from the classical function of nociceptive TRP channels in excitable cells (neurons, muscle cells and some endocrine cells) that causes an influx of ions through the cell membrane to induce a depolarization of the cell which in turn triggers action potentials [16]. Most cancer cells are classified into non-excitable cells, and overexpressed nociceptive TRP channels in cancer cells are thus unusual [17]. However, multiple roles of Ca^2+^ signaling from nociceptive TRP channels in cancer development have been independently reported. In this review, we discuss and summarize the possible mechanisms that indicate Ca^2+^ signaling from nociceptive TRP channels modulates cancer progression.

## Ca^2+^ signaling from nociceptive TRP channels potentiates initiation of cancer progression

### Nociceptive TRP channels respond to environmental stimuli and cause the aging process

Nociceptive TRP channels are predominantly expressed by distinct subsets of sensory neurons of the peripheral nervous system [18], which cause an initial response to environmental stimuli, especially microbes, chemicals or physical injuries. Although numbers of TRP channels are identified by their characteristics and functions, several TRP channels are characterized by nociceptive TRP channels, including TRPA1, TRPC1/C3/C5/C6/C7, TRPM2/M3/M8 and TRPV1/V2/V3/V4, which are involved in the nociceptive pathway [19]. Interestingly, these nociceptive TRP channels in non-excitable or excitable cells potentially initiate the aging process due to excess Ca^2+^ signaling from active nociceptive TRP channels upon continual environmental stimuli [11–15]. Environmental stimuli, such as bacterial endotoxins, oncovirus, di-(2-ethylhexyl)-phthalate (DEHP), particulate matters (PMs) or ultraviolet radiation have responding nociceptive TRP channels. The responding TRP channels of environmental stimuli was illustrated in Table 1. Some environmental stimuli (e.g., PMs) can directly activate nociceptive TRP channels [20], but others activate nociceptive TRP channels through G protein-coupled receptors (GPCR) [21]. The GPCR-TRP axis mediates sensation and inflammation responses to environmental stimuli; for instance, after irradiation by ultraviolet B (UVB), the activation of nociceptive TRP channels (e.g., TRPC7) induces response to UVB-induced skin damage through the GPCR-phospholipase C (PLC)-diacylglycerol (DAG) signaling [13]. When environmental stimuli activates the responding TRP channels, increased Ca^2+^ influx contributes to oxidative stress [22]. Mitochondria Ca^2+^ overload induces intracellular ROS accumulation and DDR, which triggers the senescence inflammation response (SIR) and senescence-associated secretory phenotype (SASP) activation, leading to genomic instability and cancer progression [23].

**Table 1.** Nociceptive TRP channels respond to environmental stimuli

**Environmental stimuli**	**Category**		**Nociceptive TRP channel**	**Reference**
Microbes	Gram-negative bacteria: bacterial endotoxins		TRPA1, TRPM3, TRPM8, TRPV1	[81]
	Oncovirus	EBV	TRPA1	[82]
		HBV	TRPC6	[83]
		HPV	TRPV4	[84]
Chemicals	Mustard oil, formalin		TRPA1	[85]
	Menthol, icilin		TRPM8	
	DEHP		TRPV1	[86]
	Particulate matter		TRPA1, TRPC6, TRPM2, TRPV1, TRPV4	[20]
Physical injuries	Mechanical gating		TRPA1, TRPC1, TRPC3, TRPC6, TRPM8	[87]
	UVA		TRPA1	[88]
	UVB		TRPC7	[13]

EBV: Epstein-Barr virus; HBV: hepatitis B virus; HPV: human papilloma virus; UVA: ultraviolet A

### Ca^2+^-activated K^+^ channels potentially trigger the cell cycle in initiating tumorigenesis

**Figure 1.**
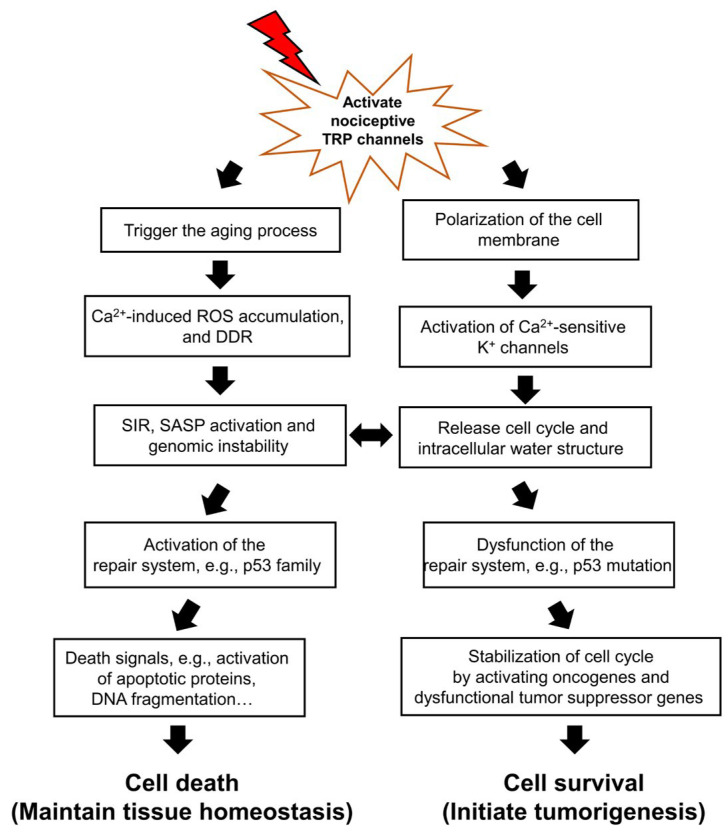
Environmental stimuli-activated nociceptive TRP channels disturb the balance between cell death and survival by high-magnitude Ca^2+^ entry. Upon the release of cell cycle arrest by initiating the aging process, the fate of cells is determined: death or survival. The repair system, such as p53 protein family is involved in this determination. When genomic instability cannot be repaired due to dysfunction of the repair system, the release of cell cycle arrest in senescent cells results in tumorigenesis, because senescent cells don’t progress to cell death. Senescent cells could initiate the cell cycle through Ca^2+^-activated K^+^ channels which are activated by nociceptive TRP channels, and stabilize the released-cell cycle with activation of oncogenes and dysfunction of tumor suppressor genes. On the other hand, in the aging process, nearly all cells face ROS released from mitochondria and DDR and SIR, and if the p53 protein family is activated, eventually contribute to cell death. Environmental stimuli-activated nociceptive TRP channels initially result in increased Ca^2+^ entry to activate death signals which oxidize and degrade proteins and induce DNA fragmentation

Excess Ca^2+^ signaling from active nociceptive TRP channels upon continuous environmental stimuli may lead to cancer progression that is due to the change of intracellular proton dynamics [24–26]. It is still unclear how Ca^2+^ signaling controls intracellular proton dynamics to initiate cancer progression and to promote tumorigenesis. Proton signaling, especially Ca^2+^ signaling, influences changes in water properties and water content in the cytoplasm that occur along with the cell cycle [27]. According to the novel theory of cancer research, the water structure between primary cells and cancer cells is completely different; the water structure in the primary cell (cell cycle arrest) is bound (like an iceberg) and molecules cannot move freely [24]. Interestingly, in cancer cells (cell cycle activation), water is free and molecules can move around easily. Proton diffusion determines if the cell physiology is faster than protein interaction [27]. Indeed, many proteins perform their functions by also being dependent on proton dynamics. In primary cells, Ca^2+^ signaling from active nociceptive TRP channels potentiates activation of Ca^2+^-activated K^+^ channels to reduce the intracellular K^+^ concentration, which releases cell proliferation by releasing the cell cycle and the intracellular water structure (Figure 1), and the free molecules, such as ions, proteins or nucleotides can move easily to maintain cell survival. This could be key to the role of Ca^2+^ in initiating tumorigenesis, because Ca^2+^ signaling from active nociceptive TRP channels requires polarization of the cell membrane and thus consequently activity of K^+^ channels, which are decisive for cell proliferation [28], similar to fertilization in triggering the development [29]. The fast proton diffusion method confirmed that fertilization induced the formation of free water from bound water [30].

Decreased intracellular K^+^ concentration in primary cells alters water structure from bound water to free water; it could be due to the K^+^ content directly affecting bound water structure. The structure of bound water is similar to an iceberg, and the melting rate of an iceberg can be determined by K^+^ content [31]. Accordingly, decreased intracellular K^+^ concentration by activation of Ca^2+^-activated K^+^ channels in primary cells may interrupt the bound water structure, similar to the melting iceberg. Furthermore, we consider how Ca^2+^ signaling from nociceptive TRP channels activates these K^+^ channels to alter water structure. TRP channels, when activated, contribute to cell depolarization via allowing Na^+^ to flow into the cell [16]. Similarly, *Xenopus* oocytes revealed a depolarization of the membrane potential by fertilization, which induces Na^+^ entry [32]. Thus, the active nociceptive TRP channels upon continuous environmental stimuli induce Na^+^ entry, which initially causes an increase in intracellular proton signaling, especially Ca^2+^ signaling; Ca^2+^-activated K^+^ channels to decrease the intracellular K^+^ concentration, then the water state is changed gradually from the bound state to the free state.

As shown in Figure 1, once Ca^2+^ signaling from active nociceptive TRP channels activates K^+^ channels to continuously release the cell cycle, the repair system quickly recovers errors to maintain tissue homeostasis. The repair system could be driven by the p53 family proteins first, and then induce death signals following resistance to tumorigenesis. The cell dynamic alternation between death and survival might be balanced until dysfunction of the repair system, especially p53 family proteins. The switch between cell death and tumorigenesis may be the SIR, which is thought to be a contributor towards tumorigenesis with genomic instability and dysfunction of the repair system [3]. Accumulation of intracellular ROS and DDR results in genomic instability and dysfunction of the repair system, followed by a change in the activity of proto-oncogenes and tumor suppressor genes to accelerate cancer development.

### Ca^2+^ signaling from nociceptive TRP channels promotes cancer progression

We pointed out that Ca^2+^ signaling from nociceptive TRP channels is involved in initiating cancer progression. More evidence of nociceptive TRP channels is shown in regulating cancer promotion, because Ca^2+^ signaling is necessary for cancer cell growth, metastasis and chemotherapy resistance [33]. The Ca^2+^ dependent activation of Ca^2+^/calmodulin-dependent protein kinase II (CaMKII) phosphorylates multiple targets, including focal adhesion kinase (FAK) which accelerates cancer cell migration and Akt, c-Jun N-terminal Kinase (JNK) and Src which promotes cancer cell proliferation [34]. CaMKII-dependent activation of hypoxia-inducible factor 1-alpha (HIF-1α) and P-glycoprotein prevents cancer cells from chemotherapy drug-induced cell death [35]. As shown in Table 2, nociceptive TRP channels are upregulated in many types of cancer cells. TRPA1 and TRPC5 perform an important role in transducing chemical nociceptive stimuli [36], but upregulated TRPA1 and TRPC5 reveal poor prognosis in cancers. TRPC1 and TRPC6 channels, which are involved in nociceptive pathways, cooperate with TRPV4 to mediate mechanical hyperalgesia and nociceptor sensitization [37]. TRPC1, TRPC6 and TRPV4 have been implicated in upregulation of breast and gastric cancer. TRPC6 also controls glioma development via regulation of G2/M phase transition [38]. TRPC3 and TRPC7 have recently been reported to be correlated with nociceptive pain in rodents [13, 39]. Although TRPC3 and TRPC7 mediate store-operated Ca^2+^ entry (SOCE) potentiating acceleration of cancer cell growth [13, 40], seldom does the study point out the function of TRPC7 in cancer development.

**Table 2.** Nociceptive TRP channels in regulating cancer progression

**Channel**	**Tumor types**	**Tumor cells *vs.* Normal controls**	**Pathological function in cancer**	**Prognosis**	**Reference**
TRPA1	Breast cancer, lung cancer, pancreatic cancer, nasopharyngeal carcinoma	Over-expression	Promote cancer cell survival against chemotherapeutic agents	Unfavorable in breast cancer, lung cancer, nasopharyngeal carcinoma	[82, 89, 90]
TRPC1	Breast cancer (PTEN-deficient type), lung cancer, gastric cancer, pancreatic cancer, colorectal cancer, glioblastoma	Over-expression	Promote cancer cell growth and metastasis	Unfavorable in breast cancer (PTEN-deficient type), gastric cancer	[91, 92]
TRPC3	Breast cancer (triple negative type), ovarian cancer	Over-expression	Promote cancer cell growth and cancer cell survival against chemotherapeutic agents	Unfavorable in breast cancer, ovarian cancer	[40, 93]
TRPC5	Breast cancer, colorectal cancer	Over-expression	Promote cancer cell survival against chemotherapeutic agents, tumor metastasis	Unfavorable in colorectal cancer	[94, 95]
TRPC6	Breast cancer, hepatoma, gastric cancer, ESCC, prostate cancer, glioblastoma	Over-expression	Promote cancer cell growth and metastasis	Unfavorable in esophageal squamous cell carcinoma	[96–101]
TRPC7	Lung cancer, skin sarcoma	Over-expression	Promote cancer cell growth	Not investigated	[13]
TRPM2	Breast cancer, lung cancer, gastric cancer, pancreatic cancer, prostate cancer, HCC, oral cancer, glioblastoma	Over-expression, mutant type in PDAC, long non-coding RNA TRPM2-AS in HCC	Promote cancer cell growth and metastasis	Unfavorable in luminal B and TP53 wild type breast cancer, lung cancer, PDAC, HCC (Long non-coding RNA TRPM2-AS)	[45, 102–107]
TRPM3	ccRCC, glioblastoma, choroid plexus papilloma	Over-expression	Promote cancer cell growth	Not investigated	[107–109]
TRPM8	Breast cancer, lung cancer, gastric cancer, ESCC, pancreatic cancer, prostate cancer, HCC, esophageal cancer, glioblastoma, neuroblastoma, urinary bladder carcinoma	Over-expression	Promote cancer cell survival against chemotherapeutic agents, cancer cell growth and metastasis	Unfavorable in urinary bladder carcinoma	[110–113]
TRPV1	Breast cancer, oral cancer, glioblastoma	Over-expression	Promote cancer cell growth	Unfavorable in breast cancer	[107, 114, 115]
TRPV2	Breast cancer, gastric cancer, ESCC, prostate cancer, HCC, ovarian cancer, oral cancer, glioblastoma, hematological cancer, urinary bladder carcinoma	Over-expression, full-length TRPV2 (f-TRPV2) in urinary bladder carcinoma	Promote cancer cell growth and metastasis	Unfavorable in multiple myeloma, ESCC	[46, 107, 111, 116, 117]
TRPV3	Breast cancer, lung cancer, oral cancer	Over-expression	Promote cancer cell growth and metastasis	Not investigated	[111, 116, 118]
TRPV4	Breast cancer, gastric cancer, pancreatic cancer, HCC, colorectal cancer, oral cancer, glioblastoma	Over-expression	Promote cancer cell growth and metastasis	Unfavorable in gastric cancer	[93, 111, 116, 119–122]

ESCC: esophageal squamous cell carcinoma; PTEN: phosphatase and Tensin Homolog deleted on Chromosome 10; PDAC: pancreatic ductal adenocarcinoma; HCC: hepatocellular carcinoma; ccRCC: clear cell renal cell carcinoma

TRPM2, TRPM3 and TRPM8 have a pathological role for a wide range of inflammatory conditions and neuropathic pain [36], and also belong to thermosensitive TRP channels [41, 42]. Those nociceptive TRPMs with overexpression facilitate malignancy in a majority of cancers (Table 2). TRPV1, nociceptor, causes pain hypersensitivity associated with neuropathic pain, peripheral inflammation [43] and cancer cell growth and metastasis (Table 2). Furthermore, long non-coding RNA, an antisense transcript of TRPM2 (TRPM2-AS), is overexpressed in prostate cancer and thought to be linked to poor prognosis [44]. The mutated TRPM2 gene also reveals a marked negative correlation with patient survival rate compared with the normal control group [45]. Upregulation of full-length glycosylated TRPV2 protein (f-TRPV2) in urinary bladder carcinoma is associated with metastatic ability, which can be regulated by short splice variant of TRPV2 (s-TRPV2). f-TRPV2 and s-TRPV2 have opposite trends of expression in cancer cells compared to normal cells [46]. Therefore, nociceptive TRP channels potentiate initiation of cancer progression and promote cancer development and malignancy.

## Epigenetic mechanisms seem to promote expression of nociceptive TRP channels in cancer cells

Ca^2+^ signaling is a regulator of pathways vital in cancer progression, enhancing cancer cell growth, metastatic ability and cell death resistance [47]. To maintain cancer development, malignant cells tend to alter expression of Ca^2+^ homeostasis genes via regulating epigenetic mechanisms [48]. Therefore, overexpression of nociceptive TRP channels in cancers is due to epigenetic regulation to sustain cancer development. We previously mentioned that active nociceptive TRP channels potentiates activation of Ca^2+^-activated K^+^ channels to reduce the intracellular K^+^ concentration, which releases cell proliferation by releasing the cell cycle and the intracellular water structure; chromosome structure could become much less tightly packed and lead to epigenetic regulation via free ions and proteins [49].

Three epigenetic mechanisms are categorized as writers, readers and erasers [50]. Writers that introduce various chemical modifications in DNA and histones, for instance, increased histone H3 acetylation of the *TRPV1* promoter region with histone acetyltransferases (HATs) resulting in upregulated levels of TRPV1 in dorsal root ganglia that ultimately induces hyperalgesia in rats [51]. Overexpression of TRPV1 is reportedly involved in both tumor growth and cancer-induced pain [52]. Indeed, the nociceptive TRP channel also induces Ca^2+^ signaling to produce a snowball effect that activates Ca^2+^ dependent transcription factors to accelerate its expression. Readers have a specialized domain containing proteins that identify and interpret those modifications. Ca^2+^ signaling from TRPV6 activates Ca^2+^-dependent calcineurin-nuclear factor of activated T (NFAT) cells which in turn influences translocation of *TRPC6* promoter to upregulate TRPC6 during pathologic cardiac remodeling [53]. TRPC6 may be overexpressed in cancers due to activating the TRPC6-NFAT pathway [54].

Erasers are the dedicated group of enzymes proficient in removing these chemical tags; most enzymes are histone deacetylase complexes (HDACs) and these play an essential role in maintaining genomic stability in cells [55]. HDACs remove acetyl groups and lead to a more closed chromatin structure, generally associated with transcriptional repression. During cancer progression, deficient HDACs activity regulates the expression and activity of numerous proteins [56]. Therefore, interruption of the histone variant macroH2A-recruited HDAC1 and HDAC2 augments overexpression of TRPC3 and TRPC6 via histone acetylation, resulting in increased cell growth and invasion in bladder cancer cells [57]. Although malignant cells potentiate intensive epigenetic regulation to upregulate nociceptive TRP channels in maintaining cancer development, the epigenetic mechanisms of nociceptive TRP channels activation in cancers needs to be further investigated.

Despite the prevalence of aging-associated cancer development, several types of cancer, especially retinoblastoma (RB), occurs mostly in the young. Epigenetic regulation of *RB* plays an important role in determining RB development, because extensive DNA methylation of *RB* promoter, *RB1* mutations and macrodeletions have been reported in RB [58, 59]. TRPV1 as well as TRPM8 are expressed in RB, and serve as prognostic factors for RB progression [60]. TRPV1-associated DNA (cytosine-5-)-methyltransferase 1 (DNMT1) activation increases methylation of genes that regulatevisceral pain sensationin the peripheral nervous system of rats [51], and it potentiates regulation of extensive DNA methylation in *RB* promoter. Besides, epigenetic modifications contribute to heritable changes in gene expression without altering the DNA sequence, but they can also lead to gene mutations [61]. Hypermethylation of genes mostly occurs as mutations in cancers, especially tumor suppressor genes or DNA repair genes [62]. It is still unknown whether TRPV1 or TRPM8 is involved in controlling germline mutations of epigenetic modifiers in RB, yet Ca^2+^ signaling from nociceptive TRP channels may influence epigenetic mechanisms in determining non-aging-associated cancer development.

## Targeting nociceptive TRP channels prohibits cancer development

**Figure 2:**
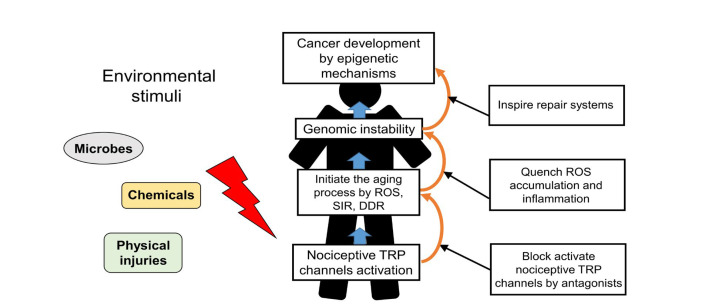
Schematic representation of nociceptive TRP channels in regulating the aging-associated cancer development and the strategic targeting of its process. Nociceptive TRP channels respond to specific environmental stimuli, such as microbes, chemicals, physical injuries, inducing excess Ca^2+^ signaling from active nociceptors upon continual environmental stimuli in the aging process. Blockage of the aging process which is induced by active nociceptive TRP channels and trigger of repair system provide a novel strategy for preventing cancer development

The role of nociceptive TRP channels in regulating cancer progression illustrates that excess Ca^2+^ signaling from active nociceptive TRP channels upon continual environmental stimuli induces the aging process and may ultimately lead to cancer progression. Consequently, blockage of excess Ca^2+^ signaling from active nociceptive TRP channels facilitates inhibition of aging-associated cancer development (Figure 2). Derinat (sodium deoxyribonucleate) protects skin against UVB-induced cellular damage and aging via inhibiting TRPCs, especially nociceptive TRPC7 [63], which reportedly mediates aging-associated tumorigenesis induced by UVB [13]. Quenching of ROS accumulation and inflammatory response by therapeutic antioxidants, such as hydrogen-rich (H_2_) water, resveratrol or sesamol significantly eliminates the aging process and thus protects against cancer development [64–66]. Although nonsteroidal anti-inflammatory drugs (NSAIDs) with anti-inflammatory effects prohibit aging-associated cancer development by inducting death signals [3], they also elicit increased ROS level in different cell types [67]. The proapoptotic accumulation of ROS in both yeast and mammalian cells is elicited by NSAIDs [68].

Cancer is an aging-associated disease. Despite blockage of active nociceptive TRP channels and ROS accumulation, the potential for cancer is not wholly eliminated. According to our pervious study, 55.8% of gene mutations occurred through the natural process of aging, and an external trigger such as environmental stimulus is required for aging-associated diseases, especially cancer [13]. DNA repair systems are inactivated and dysregulated due to genomic instability. Recently, the nucleotide precursor of nicotinamide adenine dinucleotide (NAD), nicotinamide mononucleotide (NMN), has been reported to enhance DNA damage repair and maintain mitochondrial homeostasis [69], and activates stem cells for the increase of their self-renewal [70] and the maintenance of their pluripotency [71]. Long-term administration of NMN decreases age-associated physiological degeneration in mice [72]. Similarly, mesenchymal stem cell (MSC)-derived exosomes have potential for cell-free repair for a variety of diseases and injuries through activating DNA damage repair and tissue regeneration [73]. MSC-exosomes, which carry proteins, lipids, DNA, and RNA from MSCs, have biological functions similar to MSCs, but have a smaller volume, can penetrate biofilms, have low immunogenicity, and can be stored [74].

Once cancer development is initiated by epigenetic mechanisms, cancer evolution creates malignant cells and induces changes in the genome [75]; at this stage, NMN and MSC-exosomes oppositely promote cancer progression, maintaining cancer cell growth and metastasis [76, 77]. For treating cancer cells, targeting nociceptive TRP channel activities by using multiple TRP-specific antagonists facilitates elimination or reduction of cancer development; for instance, treatment with SKF96365 and 2-aminoethoxydiphenylborate (2-APB) blocks lung cancer cell growth via inhibiting nociceptive TRPC1 [13]. Functional expression of TRPM8 in prostate carcinoma can be blocked by *N*-(4-Tertiarybutylphenyl)-4-(3-chloropyridin-2-yl)tetrahydropyrazine-1(2*H*)-carbox-amide (BCTC), clotrimazole, and DD01050 following decrease of cell growth [33]. TRPV1 activity, which is inhibited by melatonin, prevents breast cancer cells from doxorubicin-induced cell death [78]. Furthermore, because of upregulated nociceptive TRP channels in cancer cells, overactivation of TRP channels by treating their agonists results in huge ROS accumulation and induces cell death signals. Applying TRPV1 agonist capsaicin in breast cancer and glioblastoma contributes to cell apoptosis due to mitochondria Ca^2+^ overloading-increased ROS level [78–80]. But treatment with agonists of nociceptive TRP channels in several cancer cells also accelerates their malignancy and chemotherapy resistance [33].

## Conclusions

This review suggests a dual role of nociceptive TRP channels in regulating cancer progression, initiating cancer progression by aging-induced genomic instability, and promoting malignancy by epigenetic regulation. Excess Ca^2+^ signaling from active nociceptive TRP channels under continuous environmental stimuli induces intracellular ROS accumulation and DDR, which triggers the SIR and SASP activation, leading to genomic instability and cancer progression. Additionally, to maintain cancer development, cancer cells tend to alter expression of Ca^2+^ homeostasis genes via regulating epigenetic mechanisms. Nociceptive TRP channels are upregulated in many types of cancer cells and promote cancer cell growth, metastasis and chemotherapy resistance. Consequently, we propose a novel strategy for treating cancer: blockage of nociceptive TRP channels in the aging process prevents cancer initiation, and targeting nociceptive TRP channels in cancer cells provide potential therapies to prohibit cancer progression.

## Abbreviations

DDR: DNA damage response
ESCC: esophageal squamous cell carcinoma
GPCR: G protein-coupled receptors
HCC: hepatocellular carcinoma
HDACs: histone deacetylase complexes
MSC: mesenchymal stem cell
NMN: nicotinamide mononucleotide
RB: retinoblastoma
ROS: reactive oxygen species
SIR: senescence inflammation response
TRP: transient receptor potential
TRPA: transient receptor potential ankyrin
TRPC: transient receptor potential canonical
TRPM: transient receptor potential mucolipin
TRPM2-AS: antisense transcript of transient receptor potential mucolipin 2
TRPV: transient receptor potential vanilloid
UVB: ultraviolet B

## Declarations

### Copyright

© The Author(s) 2021.
